# Strong Antibacterial Polydopamine Coatings Prepared by a Shaking-assisted Method

**DOI:** 10.1038/srep24420

**Published:** 2016-04-15

**Authors:** Lei Su, Yang Yu, Yanshuang Zhao, Feng Liang, Xueji Zhang

**Affiliations:** 1 Research Center for Bioengineering and Sensing Technology, School of Chemistry and Biological Engineering, University of Science and Technology Beijing, Beijing 100083, China.; 2 The State Key Laboratory of Refractories and Metallurgy, Wuhan University of Science and Technology, Wuhan 430081, China

## Abstract

Strong antibacterial polydopamine (PDA) coatings prepared by a facile shaking-assisted method is reported for the first time. It was found that a minor modification made to the conventional synthesis procedure of PDA coatings, viz. replacing the static solution condition with a shaking solution condition by using a mechanical shaker, can produce the roughened polydopamine (rPDA) coatings at different substrates, e.g., glass, stainless steel, plastic, and gauze. The resulting rPDA coatings were characterized with Raman spectrum, zeta-potential analysis and contact angle measurement. The antibacterial activity of the rPDA coatings was evaluated by a shake flask test with gram-positive *Staphylococcus aureus*, and gram-negative *Escherichia coli* and *Pseudomonas aeruginosa* as bacteria models. Testing results revealed that, in the absence of any other antibacterial agents, the rPDA coatings exhibited remarkably enhanced antibacterial activities. In addition, such enhanced antibacterial activities of the rPDA coatings were found to be unimpaired by steam sterilization treatments.

Nowadays interest in implantation of medical devices has continued to accelerate. However, bacterial infection at the site of implanted medical devices has meanwhile become a serious ongoing problem^1^,^2^,^3^,^4^. Developing surface modification techniques to reduce the attachment of bacteria and kill the bacteria on the device surfaces is hence highly desirable^5^,^6^,^7^,^8^,^9^. Recently, contact-active antibacterial coatings have attracted increasing attention^10^,^11^,^12^,^13^,^14^,^15^. They can potentially eliminate the environment and health concerns about the biocidal agents leached from the biocide-releasing antibacterial products^14^. More encouragingly, it has been reported that common bacterial strains *Escherichia coli* (*E. coli*) and *Staphylococcus aureus* (*S. aureus*) do not develop noticeable resistance against these coatings^15^. So far, various contact-active antibacterial coatings have been proposed. Generally speaking, they need antibacterial active functional groups, e.g., quaternary ammonium compounds (QACs)^13^, tethered onto the device surfaces, or the complicated surface nanopattern structures^16^,^17^,^18^,^19^,^20^, e.g., the mimics of the wing surfaces of cicada piercing the bacteria^16^,^17^,^18^. Nevertheless, the fabrication of these coatings is usually sensitive to the surface chemistry of the materials used and many commonly used materials, e.g., metals and ceramics, have relatively inert surfaces that require harsh chemical conditions or multiple chemical steps for their pre-activation^21^.

Autoxidation of dopamine forming polydopamine (PDA) coatings has recently emerged as an extremely attractive approach for single-step surface functionalization of almost all kinds of materials. PDA also allows easily functionalization through reactions with amino- or mercapto-nucleophiles. In addition, PDA possesses biocompatible property. Therefore, PDA coatings currently have become a hot field of scientific research and technological innovation^22^,^23^,^24^,^25^,^26^, including the development of PDA-based surface modification antibacterial techniques^12^,^26^,^27^,^28^,^29^. Unfortunately, it has been demonstrated that PDA itself has only a moderate antibacterial effect^28^,^30^. As a result, in almost all antibacterial modification cases involving PDA, PDA is utilized only as a cross-linker to be pre-deposited on the surfaces of the materials, and further to tether external antibacterial agents to produce sterile surfaces^12^,^26^,^27^,^29^,^31^,^32^,^33^,^34^.

Herein, we report the shaking-assisted one-step preparation of strong contact-active antibacterial PDA coatings without using any other antibacterial reagents for the first time. It has been known that dopamine self-polymerization in a static solution can produce relatively smooth PDA coatings^23^. In this work, a simple shaking condition applied to the polymerization-coating process of dopamine is found to produce the PDA particles-roughened coatings (denoted as the rPDA coatings). More importantly, in the absence of any other antibacterial reagents, the rPDA coatings can exhibit enhanced contact-active antibacterial activities towards gram-positive *S. aureus*, and gram-negative *E. coli* and *Pseudomonas aeruginosa* (*P. aeruginosa*). In addition, such strong antibacterial performances are unimpaired by steam sterilization treatment that is a necessary procedure for implantation.

## Results and Discussion

### Preparation of the rPDA coatings

**Figure 1:**
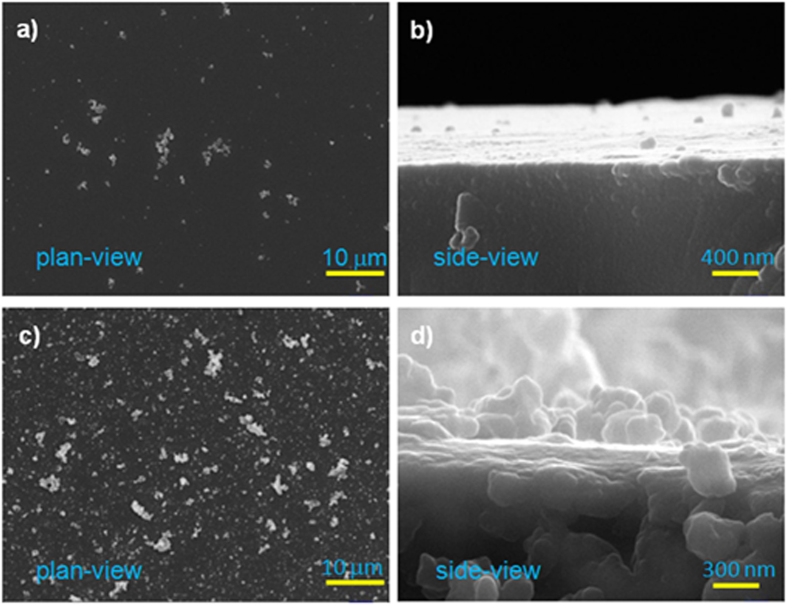
Typical scanning electron microscopy (SEM) images of the sPDA-coated (**a,b**) and rPDA-coated (**c,d**) glass substrates.

**Figure 2:**
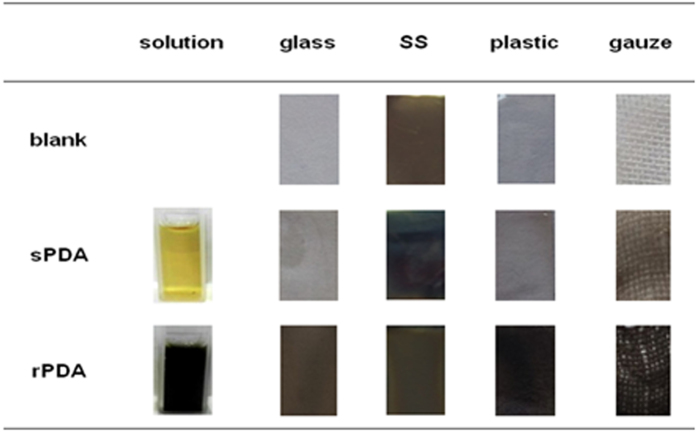
Photographs of solutions of sPDA and rPDA and substrates before (upper row) and after coating with sPDA (middle row) and rPDA (bottom row).

The preparation of the rPDA coatings follows the conventional synthesis approach of PDA coatings (e.g., at alkaline pH and O_2_ as oxidant)^23^ just with a minor modification, viz. replacing the static solution condition with a shaking solution condition by using a mechanical shaker. The effect of shaking rate on surface morphology of PDA coatings was investigated. As shown by Fig. 1a,b, the use of a relatively small shaking speed, e.g., 100 rpm, during the self-polymerization of dopamine resulted in the formation of the PDA coatings with relatively smooth surface morphology, similar to sPDA coatings formed in a static fresh dopamine solution^23^. The PDA coatings produced under static solution conditions or 100-rpm-shaking conditions are denoted as the sPDA coatings. However, the use of a shaking rate of larger than 100 rpm, e.g., 200 and 300 rpm in this study, resulted in the formation of the PDA coatings possessing relatively rough surface morphology. As shown in Fig. 1c,d, the surface of the rPDA coatings exhibited the morphology significantly different from that of the sPDA coatings, and was covered with different granules in nanosizes. The height differences of these granules were found to be too large for the ultrasmall probe tip of atomic force microscopy (AFM) to obtain the topography of the coatings. Such particles-roughened surface morphology formed under shaking conditions could be correlated with the effect of shaking conditions on the solution of self-polymerization of dopamine. It is known that during the self-polymerization of dopamine black PDA particles can be formed in the dopamine solution. We observed that in the static solution, black PDA particles settled spontaneously, leaving behind a light brown yellow but clear and transparent solution, as shown in Fig. 2; while the shaked solutions, especially as the shaking rate was larger than 200 rpm, were always turbid and black, indicating that the PDA particles remained suspended in the solution. These suspended PDA particles under the shaking condition could increase their contact probabilities with the substrates, facilitating their adhesion to the substrates and thus contributing to the formation of the rPDA coatings. In addition, the rPDA coatings, exhibiting deeper black color than that of sPDA coatings, could be formed on different substrate materials, including glass, stainless steel (SS), plastic, and gauze, as shown in Fig. 2, indicating the material-independent attributes of this approach.

### Characterization of the rPDA coatings

Before evaluating the antibacterial ability of the rPDA coatings, surface properties of the rPDA coatings were characterized by using Raman, zeta-potential analysis and contact angle measurement. Because the preparation chemistry of the rPDA coatings is essentially the same to the conventional sPDA coatings formed under static solution conditions, it is expected that the Raman features of the rPDA coatings be essentially the same to those of sPDA coatings. As expected, as shown in Fig. S1, the rPDA coatings could present the characteristic Raman peaks of PDA. They showed two obvious broad peaks: 1581 cm^−1^ and 1409 cm^−1^, which result from stretching and deformation of aromatic rings of PDA, consistent with previous studies of sPDA coatings^35^.

The measured zeta potential of the rPDA coatings was −4.58 mV at pH 7.0, also close to that of PDA as previously reported^36^. This value indicates that the overall charge of the rPDA coatings at pH 7.0 is weakly negative. However, it has been known that as for zwitterionic polymers, including proteins which can be regarded as polyampholytes, even though the overall charge of zwitterionic polymers at a pH higher than its isoelectric point is negative, positively charged groups can still exist^28^,^37^. In the case of PDA, the protonation degree of amine groups of PDA at pH 7.0 could still reach 98%, calculated according to equation 1^28^.


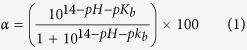


where α is the dissociation degree (protonation degree), K_b_ is the base dissociation constant and pK_b_ of amine groups of PDA was 5.3^28^. It is known that positively charged functional groups can cause bacteria lysis by contacting the cell wall of bacteria. Therefore, these protonated amine groups might provide the basic antibacterial activity of PDA itself.

**Figure 3:**
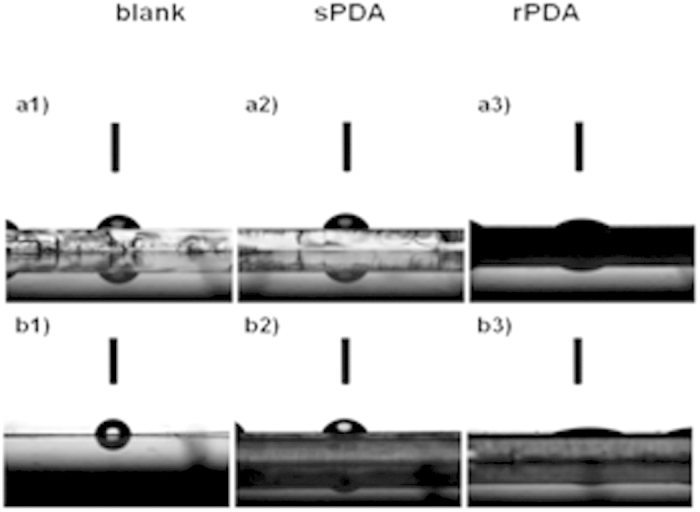
Typical static contact angles of bare (a1, b1), sPDA-coated (a2, b2), and rPDA-coated (a3, b3) glass (a1–3) and SS (b1–3) substrates.

On the other hand, contact angle measurement revealed an abnormal phenomenon that the rPDA coatings could exhibit obviously different wettability from the sPDA coatings. As shown in Fig. 3, bare substrates, e.g., glass (a1) and SS (b1), exhibited static contact angles of ~54^o^ and ~82^o^, respectively. After sPDA coating, the static contact angles changed to ~59^o^ (a2) and ~60^o^ (b2). The converged contact angles were consistent with the published values of 50–60^o^ for sPDA coatings^38^. However, as shown in Fig. 3, the rPDA coatings formed on both of the substrates presented dramatically decreased contact angles of less than 15^o^ (a3, b3), indicating more hydrophilic surfaces. Evidently, the rough surface morphology of the formed rPDA coatings strongly affected the apparent contact angle. The low values of the apparent contact angles may be associated with the tendency of water droplets to immerse through the gaps between PDA particles and aggregates. This phenomenon is somewhat akin to that observed at the nanostructured coatings, for instance, the surface nanostructures of the ZnO nanorods-based films can also exert a remarkable influence on the wettability^10^.

### Antibacterial activity of rPDA coatings

**Figure 4:**
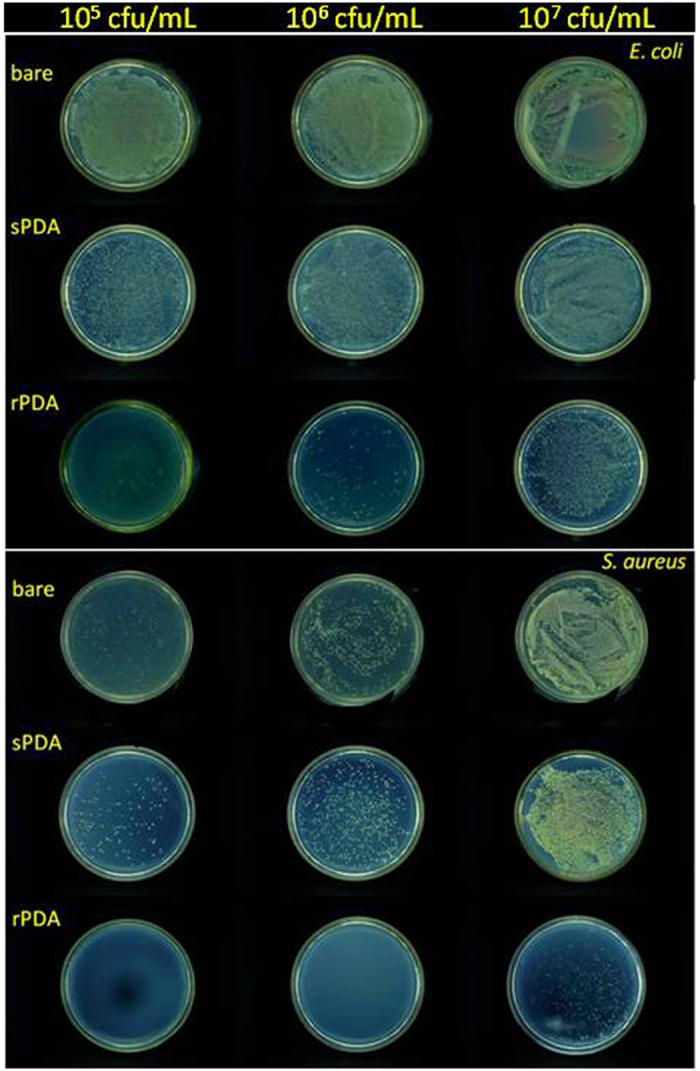
Typical photographs of the agar plate testing results of bare substrate, the sPDA and the rPDA coatings towards *E. coli* and *S. aureus* with different concentrations.

The antibacterial properties of the rPDA coatings were evaluated by a shake flask test according to ASTM E2149–01, a quantitative antibacterial test method for determining the antibacterial activity of non-leaching antibacterial agents under dynamic contact conditions^39^. As a comparison, the antibacterial properties of the sPDA coatings were also evaluated with the same protocol. In recent studies, antibacterial activity of PDA coatings has been investigated and the conclusion that PDA coatings exhibit a moderate antibacterial effect has been made^28^,^30^,^40^. In the present study, the sPDA coatings were also observed to exhibit only a low antibacterial effect on *E. coli* and *S. aureus*. The bactericidal ratios are shown in Table 1. Typically, at a bacteria concentration of 10^5^ colony-forming unit (CFU) mL^−1^, after 18–24 hour incubation, the bactericidal ratio of the sPDA coatings toward *E. coli* and *S. aureus*, respectively, was less than 5%. Due to that the colonies on the agar plate corresponding to antibacterial activities of the sPDA coatings and bare substrates were too dense to be exactly counted, the agar plate testing results were directly showed in the form of photographs (Fig. 4). As revealed, the colonies on the agar plate were dense, indicating that the sPDA exhibited relatively moderate antibacterial activity, in consistence with previous studies^28^,^30^,^40^. In addition, gram-negative *P. aeruginosa* is the most common cause of infections of burn injuries, and is also one of the most frequent colonizer of medical devices (e.g., catheters)^41^. Therefore, the antibacterial activity of the coatings towards *P. aeruginosa* was also evaluated. From Table 1, it can be seen that the bactericidal ratio of the sPDA coatings toward *P. aeruginosa* was low. Typically, at a bacteria concentration of 10^5^ CFU mL^−1^, after 18–24 hour incubation, the bactericidal ratio of the sPDA coatings toward *P. aeruginosa* was less than 5%.

However, unexpectedly, the rPDA coatings were found to possess significant killing efficiencies. As shown in Table 1, all of the bactericidal ratios of the rPDA coatings were remarkably high, e.g., close to 100% for three kinds of bacteria at a typical bacteria concentration of 10^5^ CFU mL^−1^, comparable with the classical QACs and Ag^+^. Even at the used highest bacteria concentration, i.e., 10^7^ CFU mL^−1^, after 18–24 hour incubation, the bactericidal ratios of the rPDA coatings toward three kinds of bacteria still reached more than 90%. The bactericidal results were also directly visualized from the photographs of the agar plates (Fig. 4). These results were remarkably different from those of the sPDA coatings, indicating that the rPDA coatings possess enhanced antibacterial activity as compared with the sPDA coatings. Moreover, we found that the rPDA coatings still maintained excellent antibacterial ability after storage in deionized water without any specific care at room temperature for at least 10 days (Fig. S2). On the other hand, harsh conditions such as ultrasonication operation were used to destroy the integrity of the rPDA coatings, detaching the PDA particles from the coatings. We then collected these detached PDA particles to evaluate their antibacterial effect, and found a moderate antibacterial effect, as shown in Figs S3 and S4. Therefore, the enhancement of antibacterial ability exhibited by the rPDA coatings should be associated with the surface roughening on the rPDA coatings. Surface roughening can increase surface irregularities, which have been reported to be propitious to the landing and accumulation of free-swimming bacteria^42^. In this context, the surface roughening of the rPDA coatings could increase the contact between PDA and bacteria, leading to the enhanced killing efficiency.

**Figure 5:**
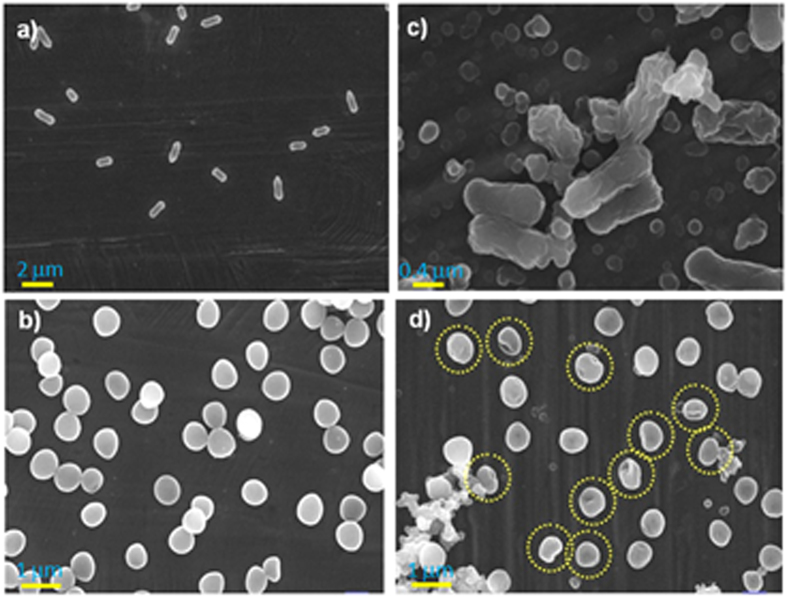
Typical SEM images of the bacteria before (**a,b**) and after (**c,d**) being treated with rPDA coatings. *E. coli*: (**a,c**); *S. aureus*: (**b,d**).

The mechanism of cell death caused by contact-active antibacterial coatings is commonly associated with the damage of cell surfaces^16^,^43^,^44^. To test this hypothesis, SEM was used to compare the surface morphologies of both the native and the rPDA coatings-treated *E. coli* and *S. aureus*. Prior to SEM observations, the blank and rPDA-coated SS slides were incubated respectively with the cell suspensions of 10^5^ CFU mL^−1^ for a short incubation time, e.g., 5 hours, then were treated with 2.5% glutaraldehyde for cells fixation, followed by ethanol (20–100%) dehydration and air-drying. As revealed by SEM observations (Fig. 5a,b), on the blank substrates incubated with the cell suspensions the fixed cells had unchanged shapes. However, on the rPDA-coated substrates incubated with the cell suspensions few cells could be found. Therefore, the cell suspension with a higher concentration, 10^8^ CFU mL^−1^, was used to incubate the rPDA-coated SS slide for 5 hours for preparing the fixed cells for SEM observation. As shown in Fig. 5c,d, relatively more bacteria could be found on the rPDA-coated substrates. Careful observation of these fixed bacteria could reveal that the *E. coli* cells treated with the rPDA coatings were significantly changed and showed major damage, with a loss of the intact rod-like shape, indicating damage to the cell membrane. Similarly, the *S. aureus* cells treated with the rPDA coatings shrunk and lost its spherical shape (marked in yellow circles), indicating possible damage to cell membrane.

In addition, it is well-known that all materials implanted within the body or placed in contact with corporeal fluids must be sterilized, and the preferred sterilization process is high temperature steam sterilization^45^. However, the steam sterilization treatment may impair seriously the performances of polymeric functional materials. For example, Rao and Sharma found that the antibacterial chitosan films underwent degradation when autoclaved at 121 °C for 15–30 min^46^. Herein, the effect of steam sterilization treatments on the antibacterial activity of the rPDA coatings was evaluated. The rPDA coatings were subjected to steam sterilization at 121 °C for 30 min and then their antibacterial activity against *E. coli* and *S. aureus* was measured respectively. Steam sterilization and subsequent incubation process were repeated twice. Antibacterial results are summarized in Table 2. It can be seen that the rPDA coatings exhibited unaffected antibacterial activity even after high temperature steam sterilization at 121 °C twice. Such unimpaired antibacterial activity by steam sterilization treatments will facilitate the practical application of the rPDA coatings.

Furthermore, the biocompatibility of the rPDA coatings was evaluated by examining the viability of the adherent mammalian cancer cell line, HeLa cells, incubated with the rPDA coatings. No qualitative differences in the attached cells were observed by microscopy, as shown in Fig. S5. These results confirmed the good biocompatibility of the rPDA coatings^47^. These advantageous antibacterial and biocompatible properties of the rPDA coatings indicate a promising future for the rPDA coatings as the candidate for the use in biomedical fields, for instance, to inhibit implant infections.

## Conclusion

This study has demonstrated that a shaking condition applied to the self-polymerization of dopamine in alkaline solution can facilitate the formation of the rPDA coatings. The rPDA coatings exhibited remarkably enhanced antibacterial activities against gram-positive *S. aureus*, and gram-negative *E. coli* and *P. aeruginosa*. The damage of the cell membranes of bacteria incubated with the rPDA coatings supported the contact-kill mechanism. In addition, the antibacterial property of the rPDA coatings was found to be unimpaired by steam sterilization treatments. The rPDA coatings thus represent an excellent approach for substrate material-independent, single-step, green, antibacterial modification to fabricate contact-active and biocompatible antibacterial polymer coatings.

## Methods

### Materials

Dopamine hydrochloride was purchased from Sigma-Aldrich. Tri(hydroxymethyl) amino methane (Tris)-HCl, tryptone, yeast extract, agar powder and other chemicals of at least analytical reagent were obtained from Sinopharm Chemical Reagent Co., Ltd. (Beijing, China). *S. aureus* (ATCC 29213) and *E. coli* (ATCC 8739) were obtained from China General Microbiological Culture Collection Center (Beijing, China). Human cervix carcinoma cells (HeLa) were obtained from China Infrastructure of Cell Line Resources (Beijing, China). Aqueous solutions were prepared using deionized water (Milli-Q system).

### Preparation of the PDA coatings

Substrate materials including glass, stainless steel, plastic, and gauze (ca. 2.5 cm ×1 cm) were immersed in the freshly prepared dopamine solution (2 mg mL^−1^ dopamine, 25 mM Tris-HCl buffer solution, pH 8.5) for 24 h at 37 °C and various shaking rates using an incubator shaker (SPH-111 F, Shanghai, China). Then, the substrates were taken out, rinsed using deionized water, and dried in air.

### Antibacterial activity testing

*S. aureus*, *E. coli* and *P. aeruginosa* were used as test organisms. A colony of the bacteria grown on a LB agar plate was used to inoculate 5 mL of LB broth in a sterile 50 mL conical tube (Falcon). The culture was incubated at 37 °C and 200 rpm for 18–20 h. Then, the incubated test culture was diluted using a sterilized phosphate buffer (pH 7.0) to the desired concentration. Antibacterial activity of the PDA-modified substrate materials and blank control substrate materials (2.5 × 2.0 cm^2^) were evaluated according to the ASTM E2149–01, which is a quantitative antibacterial test method for determining the antibacterial activity of immobilized antibacterial agents under dynamic contact conditions. For testing the effect of steam sterilization on the antibacterial activity of the rPDA coatings, the rPDA coatings on glass slides were treated with high-pressure steam sterilizer at 121 °C for 15 min. These materials were respectively incubated with 4 mL of cell suspension in a 5 mL conical tube at 37 °C and 200 rpm. Samples were taken after 24 h, and plated in nutrient agar. The inoculated plates were incubated at 37 °C for 24 h, and the surviving cells were counted. The experiments were performed in triplicate. Antibacterial testings towards *P. aeruginosa* (ATCC 27853) were performed in the Antimicrobial Material Testing Center of Technical Institute of Physics and Chemistry, Chinese Academy of Sciences (Beijing, China). The antibacterial activity was expressed as R = % reduction of the organism after contact with the test substrate materials compared to the number of bacterial cells surviving after contact with the control.

### Cell morphology observation

For SEM observation, the rPDA-coated stainless steel slides were incubated with the cell suspensions of *S. aureus* and *E. coli*, respectively, at 37 °C and 200 rpm. Blank stainless steel slides were used as the control. The slides were taken out after 5 h. The bacteria were fixed in 2.5% glutaraldehyde for 2 h and rinsed carefully with distilled water. Then, the bacteria-modified slides were dehydrated with ethanol (20–100%), air-dried, and sputter-coated with carbon for SEM observation.

### Cytotoxicity assay

HeLa cells were maintained in Dulbecco’s modified Eagle media (DMEM) containing 10% (v/v) fetal bovine serum (FBS) at 37 °C in a humidified atmosphere of 5% CO_2_. The cells were harvested using 0.25% trypsin, counted, and resuspended in the cell culture media at a concentration of 5 × 10^4^ cells mL^−1^. 2 mL of this suspension was seeded in the wells of a 6-well plate containing the rPDA-coated and blank substrates, respectively, and incubated for 24 h at 37 °C in a humidified atmosphere of 5% CO_2_. The amount of cells attached to the substrates was evaluated by optical microscopy (TS100, Nikon, Japan).

### Apparatus

Raman spectra were obtained at room temperature on a Raman system (JY-HR 800). Surface morphologies of samples were characterized with a ZEISS Supra 55 FE-SEM. Contact angles were measured at room temperature on an OCA20 system (Data-Physics, Germany). The average contact angle values were obtained by measuring at five different positions on the same sample. Zeta-potential measurement was conducted on a Malvern Zetasizer Nano ZS90.

## Additional Information

**How to cite this article**: Su, L. *et al.* Strong Antibacterial Polydopamine Coatings Prepared by a Shaking-assisted Method. *Sci. Rep.* **6**, 24420; doi: 10.1038/srep24420 (2016).

## Supplementary Material

Supplementary Information

## Figures and Tables

**Table 1:** Bactericidal ratios of the sPDA and rPDA coatings measured by the shaking flask test.

Bacteria Samples	*E. coli* concentration CFU mL^−1^			*S. aureus* concentration/CFU mL^−1^			*P. aeruginosa* concentration/CFU mL^−1^		
	10^5^	10^6^	10^7^	10^5^	10^6^	10^7^	10^5^	10^6^	10^7^
sPDA coatings	≤5	≤5	≤5	≤5	≤5	≤5	≤5	≤5	≤5
rPDA coatings	≥99	≥99	≥90	100	≥99	≥95	100	≥99	≥95

**Table 2:** Effect of steam sterilization on the bactericidal ratio of the rPDA coatings.

Bacteria Cycle numbers of steam sterilization treatment	*E. coli* concentration CFU mL^−1^			*S. aureus* concentration/CFU mL^−1^		
	10^5^	10^6^	10^7^	10^5^	10^6^	10^7^
1	≥99	≥99	≥90	100	≥99	≥95
2	≥99	≥99	≥90	100	≥99	≥95
