# Supplementary material for: Strong Antibacterial Polydopamine Coatings Prepared by a Shaking-assisted Method
Source: Sci Rep. 2016 Apr 15;6:24420. doi: 10.1038/srep24420 (PMC4832207; doi:10.1038/srep24420)
Supplement: Supplementary Information [file srep24420-s1.pdf]

Supplementary Information

Strong Antibacterial Polydopamine Coatings Prepared by a Shaking-assisted Method

Lei Su,[1,*] Yang Yu,[1] Yanshuang Zhao,[1] Feng Liang,[2] Xueji Zhang[1]

[1] Research Center for Bioengineering and Sensing Technology, School of Chemistry and Biological Engineering, University of Science and Technology Beijing, Beijing 100083, China. E-mail: sulei@ustb.edu.cn

[2] The State Key Laboratory of Refractories and Metallurgy, Wuhan University of Science and Technology, Wuhan 430081, China.

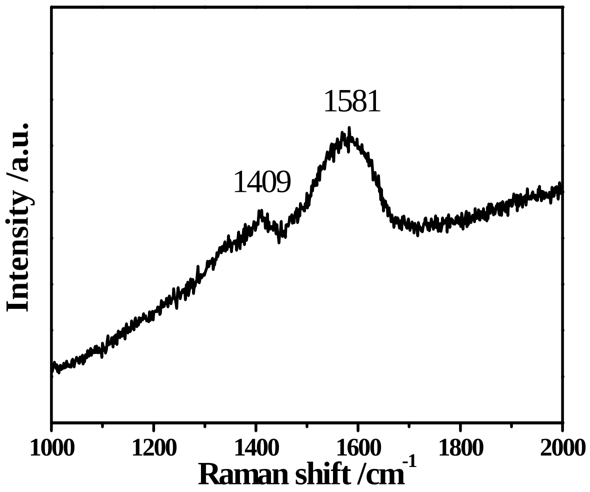

Fig. S1. Raman spectrum of the rPDA films.

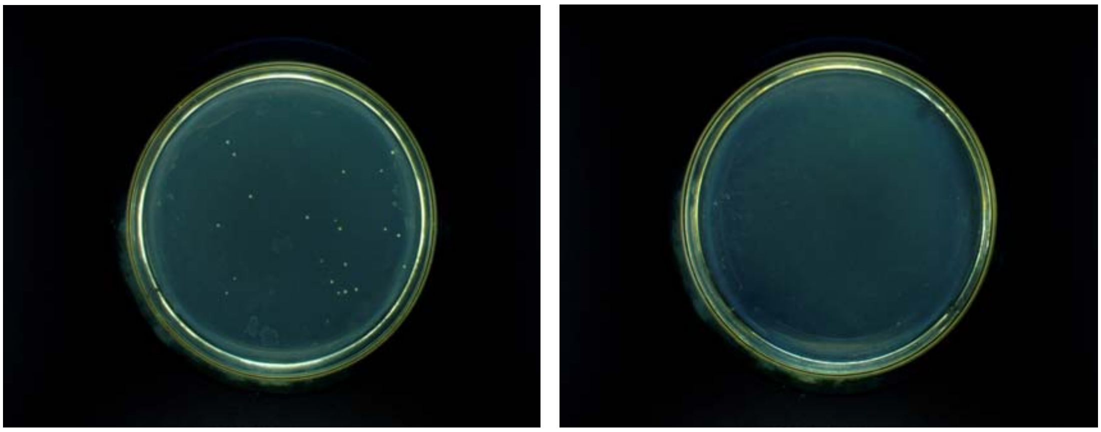

Fig. S2. Typical photographs of the agar plate testing results of rPDA films towards *E. coli* (left) and *S. aureus* (right) of 10^5 CFU mL^{-1} after storing the rPDA in deionized water at room temperature for 10 days, followed by incubation with *E. coli* and *S. aureus* of 10^5 CFU mL^{-1}, respectively.

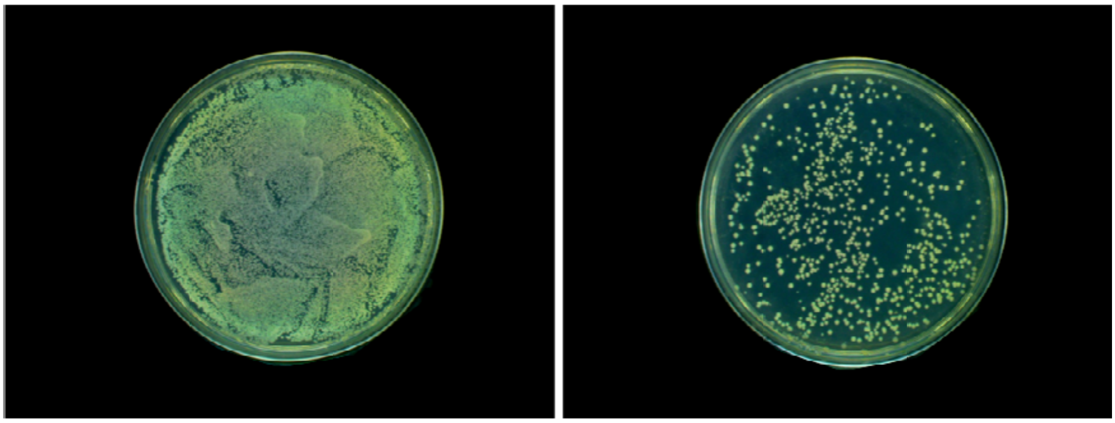

Fig. S3. Typical photographs of the agar plate testing results of bare and PDA particles detached from the rPDA films towards *E. coli* (10^5 CFU mL^{-1}).

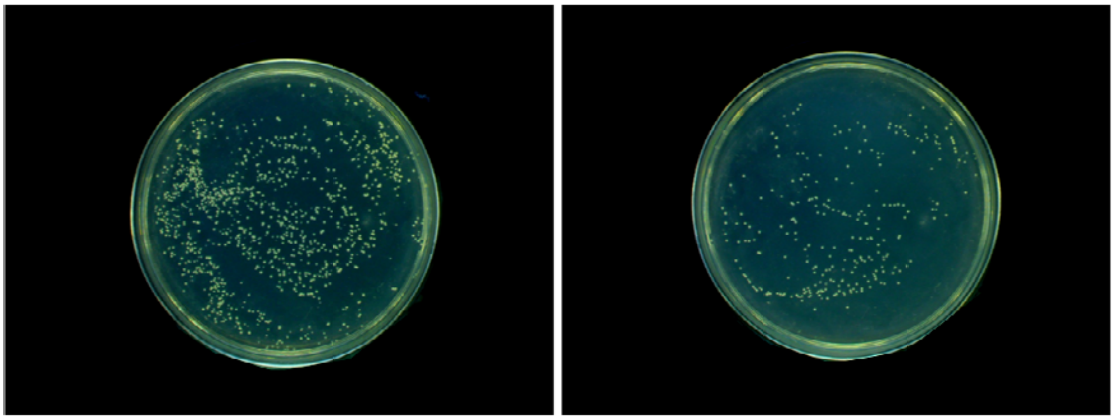

Fig. S4. Typical photographs of the agar plate testing results of bare and PDA particles detached from the rPDA films towards *S. aureus* (10^5 CFU mL^{-1}).

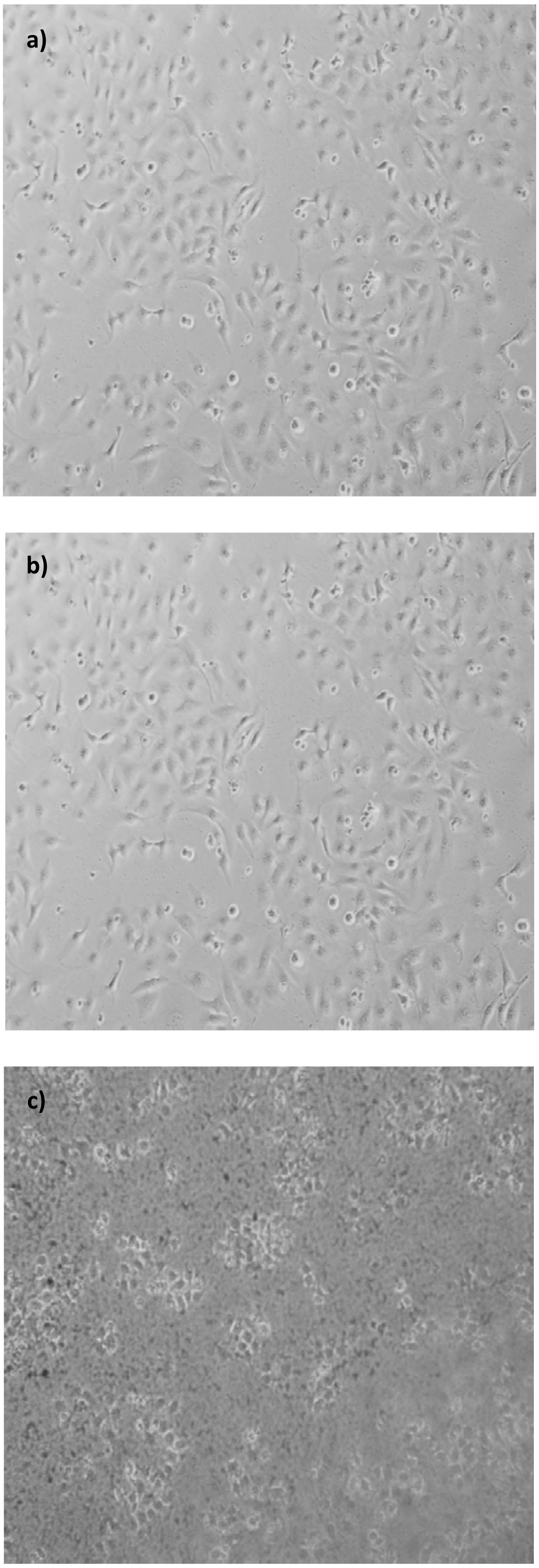

Fig. S5. Microscopic pictures of HeLa cells after incubation with bare (a), sPDA (b) and rPDA (c) films modified glass substrates.
